# An iPSC-derived model for drug screening in cerebral small vessel disease

**DOI:** 10.1177/0271678X251389379

**Published:** 2025-11-07

**Authors:** Alessandra Granata, Maha Al-Thani, Mary Goodwin-Trotman, David M Smith, Tom Van Agtmael, Hugh S Markus

**Affiliations:** 1 Stroke Research Group, Department of Clinical Neurosciences, University of Cambridge, Cambridge, UK; 2 Hit Discovery, Discovery Sciences, R&D, AstraZeneca, Cambridge, UK; 3 School of Cardiovascular and Metabolic Health, University of Glasgow, Glasgow, UK

**Keywords:** Cerebral small vessel disease, vascular dementia, induced pluripotent stem cells, drug screening, matrix metalloproteinases

## Abstract

Despite the enormous health importance of cerebral small vessel disease (cSVD) there are few treatments available. A major limitation in screening new therapies is a lack of clinically relevant disease models. Induced pluripotent stem cell (iPSC) technology allows creation of in vitro models of the vascular unit, which is found to be affected in cSVD. Here we derived iPSC from skin biopsies obtained from patients with two of the most common monogenic forms of cSVD, HTRA1 mutations and COL4A1/2 mutations. iPSCs were differentiated into brain endothelial-like cells and mural cells and co-cultured in a transwell system to replicate the vascular unit. iPSC models derived from the two distinctive forms of monogenic cSVD demonstrated a common molecular phenotype characterised by tight junction defects, elevated actin stress fiber expression and mural cell loss, increased blood–brain barrier (BBB) permeability and elevated matrix metalloproteinase (MMP) levels. Moreover, the model was successfully optimised to a 96-well format to screen 13 MMP inhibitors, three of which effectively restored permeability to control levels. Our findings provide evidence that MMP inhibition could serve as a potential therapeutic strategy for cSVD as well as present a robust model to screen drugs in cSVD.

## Introduction

Cerebral small vessel disease (cSVD) causes a quarter of all ischaemic strokes (lacunar stroke), is the leading cause of intracerebral haemorrhage, and is the most common pathology underlying vascular cognitive impairment (VCI) and vascular dementia.^1^ It is characterised by pathological changes in the small penetrating cerebral arteries and capillaries that supply the brain white matter and deep grey matter nuclei. These changes are evident on neuroimaging (MRI) as white matter hyperintensities, lacunar infarcts and cerebral microbleeds.^2^ Despite its enormous global health burden, therapeutic approaches for cSVD are limited to risk factor modifications, such as anti-hypertensive therapy, and no specific disease modifying therapies are available.^3^ A major reason for the lack of effective therapies has been an incomplete understanding of the underlying molecular disease mechanisms. Recent advances in genomics, and in experimental models, have identified several promising therapeutic targets, but translating these into studies in humans is challenging. A particular problem is screening novel therapies in experimental studies prior to human trials. There are no ex vivo models available, while animal models have significant limitations including a lack of similar end organ damage to that seen in humans, and the high cost and time required for screening multiple therapies.^4,5^ This highlights the urgent need for clinically relevant in vitro models which allow for testing of potential treatments with a clinically meaningful readout.

A key emerging theme from recent studies is a central role of neurovascular unit (NVU) disruption.^6^ The NVU is a highly complex structure in the central nervous system, consisting of brain endothelial cells, mural cells, astrocytes and a basement membrane made of extracellular matrix (ECM) proteins produced by these cells. The NVU is essential for maintaining the integrity of the blood–brain barrier (BBB), which regulates the passage of substances in and out of the brain.

The importance of NVU disruption, particularly involving the ECM and its associated proteins, was initially highlighted in the rare monogenic forms of cSVD caused by mutations in *HTRA1* (CADASIL2), *COL4A1/2* (Gould syndrome) and *NOTCH3* (CADASIL).^7^ In particular, disruption of the ECM and its associated proteins (matrisome) have been implicated.^8^ Both *COL4A1/2* and *HTRA1* mutations are linked to defective ECM regulation within mural cells: *COL4A1/2* mutations impair basement membrane assembly,^9,10^ while *HTRA1* encodes a secreted serine protease involved in ECM degradation and regulation of TGF-β signalling.^11^ Mutations in *HTRA1* reduce proteolytic ECM degradation due to loss of protease activity, leading to impaired ECM turnover and dysregulated TGF-β signalling.^12^ These ECM disruptions likely contribute directly to mural cell dysfunction and degeneration, which in turn exacerbates BBB breakdown. More recently genetic and experimental studies have identified similar processes in the more common sporadic polygenic cSVD, with common variants in genes (i.e. *COL4A2* and *HTRA1*) underlying NVU function associated with disease risk.^13,14^ Consistent with the importance of NVU dysfunction, increased BBB permeability has been reported both neuropathologically,^15^ and on MRI,^16^ in humans with cSVD.

This suggests that a model capturing these key processes, and in particular NVU and matrisome dysfunction, could facilitate the screening of potential therapies for cSVD. Ideally, such a model would support the screening of compound libraries. Cellular models are being increasingly used to model BBB function with a readout including BBB permeability. While most models to date have relied on generic cell lines, the use of induced pluripotent stem cell (iPSC) technology allows for the development of models using cells derived from patients with specific genetic variants that cause cSVD.

Here, we have used cells from patients with monogenic forms of cSVD (*HTRA1* and *COL4A1/2* mutations), transforming them into endothelial cells and mural cells to create a cSVD in vitro model. We demonstrate that models from patients with these genetic causes of cSVD share a common phenotype including increased permeability and loss of mural cells. Furthermore, based on evidence implicating matrix metalloproteinases (MMP) in cSVD^10^ we show that this model can serve as platform to evaluate new therapies by using it in a proof-of-concept screen of MMP inhibitors.

## Material and methods

### Generation of human iPSC cell lines

We established four iPSC lines from skin biopsies taken from patients with monogenic cSVD (Table 1). All had recognised disease-causing mutations and the clinical phenotype of monogenic cSVD. They included two with autosomal dominant *HTRA1* mutations (R166H and P331L) and two with autosomal dominant *COL4A1* (G755R^17^) and *COL4A2* (G702D^18^) mutations^10^ (Figure S1(a)). All patients gave written informed consent, and the study was conducted in accordance with the Declaration of Helsinki and approved by the Health Research Authority (East of England – Cambridge Central Research Ethics Committee; REC reference ref: 16/EE/0118) on November 9, 2023.

**Table 1.** iPSC lines.

Name	Gene	Mutations	Pathology	Age	Sex	Clinical features	Reprogramming method and source
WT1 (SOJD_3)	–	–	Healthy control	45–49	F	–	Sendai virus; HIPSCI Consortium
WT2 (WIBJ_2)	–	–	Healthy control	55–59	F	–	Sendai virus; HIPSCI Consortium
WT3 (EIPL-1)	–	–	Healthy control	40–44	F	–	Sendai virus; HIPSCI Consortium
WT4 (SERU_7)	–	–	Healthy control	65–69	F	–	Sendai virus; HIPSCI Consortium
COL4A1	*COL4A1*	G775R	SVD patient	65	F	Migraine; WMH; stroke^17^	Sendai virus; hiPSC Core Facility (Cambridge)
COL4A2	*COL4A2*	G702D	SVD patient	75	M	Asymptomatic^18^	Sendai virus (Glasgow)
HTRA1 line 1	*HTRA1*	R166H	SVD patient	71	F	Stroke	Sendai virus; hiPSC Core Facility (Cambridge); Table S1
HTRA1 line 2	*HTRA1*	P331L	SVD patient	59	F	Migraine; encephalopathy; cognitive impairment	Sendai virus; hiPSC Core Facility (Cambridge); Table S1

We also used two types of control lines: four commercially available healthy (WT) iPSC lines (Table 1); we created isogenic gene edited iPSC clones for the *COL4A1/A2*^13^ and *HTRA1* mutations, reverting the mutation to the normal ‘unaffected’ variant (Figure S1(a) and Table S2).

### Human iPSC culture

All hiPSC lines were cultured in TeSR™-E8 media (STEMCELL Technologies) or E8 media (Dulbecco’s Modified Eagle Medium/nutrient mixture F-12 (DMEM/F-12)) with insulin–transferrin–selenium (Thermo Fisher Scientific), sodium bicarbonate (Thermo Fisher Scientific) and L-ascorbic acid (Merck) supplemented with FGF2 (4 µg/mL; Biochemistry Department, University of Cambridge) and TGF- β1 (1.74 µg/mL; R&D Systems) using Vitronectin XF (STEMCELL Technologies) as chemically defined xenofree cell culture matrix. HTRA1 line 1 and line 2 hiPSC lines were characterised and validated for pluripotency markers expression by immunostaining and qPCR (Figure S1(a) and (b)) and by formation of the three germ-layers (Figure S1(c)) by the Cambridge Biomedical Research Centre iPSC core. Karyotype processing was performed by Medical Genetics Service at the Cambridge University Hospital, on 20 fixed iPS cell suspension at passage 32. The preparations obtained were of sufficient quality to detect numerical and large structural abnormalities.

All hiPSC were routinely tested for presence of mycoplasma contamination by Mycoplasma Experience Ltd. HiPSC were differentiated into both mural cells and endothelial cells.

### HiPSC differentiation into mural cells

hiPSC were driven to an intermediated stage of neural crest (NC) differentiation. hiPSC were detached from vitronectin coated plates using ReLeSR (STEMCELL Technologies) as previously described.^19,20^ Clumps were plated at a density of 300 in 0.1% gelatin-coated six-well plates in CDM–polyvinyl alcohol (PVA) for 4 days without splitting. CDM was composed of Iscove’s modified Dulbecco’s medium plus Ham’s F12 NUT-MIX (Thermo Fisher Scientific) medium in a 1:1 ratio, supplemented with chemically defined lipid concentrate (Thermo Fisher Scientific), transferrin (Roche Diagnostics), insulin (Roche Diagnostics) and monothioglycerol (Sigma–Aldrich) supplemented with FGF2 (12 ng/mL; R&D Systems) and SB-431542 (10 mmol/L; Tocris), referred as FSB. At day 4, hiPSC was dissociated using TrypLE Express (Thermo Fisher Scientific) and seeded as single cells at a 1:3 ratio on 0.1% gelatin-coated plates in FSB. NC cells were passaged every time reached confluence, up to 12 passages.

For mural cells (MC) differentiation, NC cells were dissociated using TrypLE Express and cultured in MC differentiation medium (CDM–PVA supplemented with PDGF–BB (10 ng/ml; Peprotech) and TGF-β1 (2 ng/ml; Peprotech) for 12 days. MCs were expanded in serum replacement containing media with 0.25 mM L-ascorbic acid for up to 2 weeks.

### HiPSC differentiation into BEC

hiPSCs were differentiated to brain microvascular endothelial-like cells (BEC) as previously described, with modifications.^21^ hiPSCs were washed once with 1× PBS (Corning^®^), dissociated with StemPro™ Accutase™ Cell Dissociation Reagent (Thermo Fisher Scientific) for 4 min, and collected by centrifugation. hiPSCs were then resuspended in E8 medium containing 10 μM Y27632 (Tocris Bioscience) and seeded onto Matrigel-coated six-well plates at a density of 1.56 × 10^4^/cm^2^. The following day, the cells were switched to TeSR™-E6 medium (STEMCELL Technologies) to initiate the differentiation. Media was changed every day for 4 days. On day 5, the cells were switched to endothelial media (EC), which consisted of a basal human endothelial serum-free media (SFM; Thermo Fisher Scientific), supplemented with B27 (Thermo Fisher Scientific), basic fibroblast growth factor (bFGF; 10 ng/ml; R&D Systems) and all-trans retinoic acid (RA; 10 μM; Sigma–Aldrich). Cells were then left to incubate for 48 h in EC medium without a media exchange. On day 6, resultant BEC cells were washed with PBS and dissociated with accutase to single cells and plated at a density of 2.5 × 10^5^/well on 24-well coated with collagen IV (from human placenta, 1 mg/ml, Bornstein and Traub Type IV, C5533; Sigma–Aldrich) and fibronectin (from bovine plasma, 1 mg/ml, F1141; Sigma–Aldrich). Twenty four hours after plating, media was refreshed to EC medium without bFGF and RA. Subsequent media changes were performed every 2 days for 6 days.

### CRISPR-mediated gene editing

To generate the isogenic lines, a CRISPR-gene editing method was performed using single guide synthetic RNA (sgRNA; Synthego), SpCas9 protein (Biochemistry Department, University of Cambridge) and a 90-nt single-stranded oligodeoxynucleotide (ssODN; IDT) for homology-directed repair. To avoid ssODN cleavage by Cas9, a silent mutation was introduced in the NGG codon upstream of the correction site. For gene targeting, 200,000 cells were electroporated with Cas9/sgRNA together with ssODN using the Amaxa 4DNucleofector CA-137 program code (Lonza). Transfected cells were plated onto vitronectin coated-plates in TeSR™-E8 media with 10 μM Y-27632 and CloneR (STEMCELL Technologies). After 48 h, the pool of transfected cells was sequenced to test recombination efficiency. Positive clones were selected by serial dilution and manual selection and sequenced by sanger sequencing (Table S2).

### Quantitative real-time PCR

Complementary DNA (cDNA) was synthesised from 250 ng total RNA using the Maxima First Strand cDNA Synthesis Kit (Thermo Fisher Scientific). Quantitative real-time polymerase chain reaction (PCR) mixtures were prepared with the FAST-SYBR Green Master Mix (Thermo Fisher Scientific) and analysed using the QuantStudio 7 Flex (Applied Biosystems and Thermo Fisher Scientific). Data are expressed as fold change (ΔΔCT) relative to the mean of GAPDH and PBGD housekeeping genes. Primer sequences are listed in Table S3.

### Functional and pathway enrichment analysis

Functional and pathway enrichment analysis of the DEGs from bulk RNA sequencing data^13^ was performed using Metascape platform (http://metascape.org/gp/index.html).

### Immunofluorescence staining and quantification

Adherent cells were fixed using 4% PFA (Boster) for 5 min at RT (hiPSC and MC) or 100% ice cold methanol (BEC) for 15 min at −20 °C and then washed three times with 1× PBS containing calcium and magnesium (Oxoid). Cells were permeabilised with 0.05% Triton X-100 (Sigma–Aldrich) in PBS and blocked with PBS +3% BSA or 10% FBS for 60 min at RT. Primary antibodies (1:200; Table S4) incubations were performed at 4 °C overnight and Alexa Fluor tagged secondary antibodies (1:400; Molecular Probes Invitrogen) and DAPI (Sigma–Aldrich) applied for 1 h at room temperature the following day. Images were acquired on a Zeiss LSM 700 confocal and analysed with Fiji–ImageJ software.

For tight junction quantification in hiPSC–BEC, following immunostaining with occludin or claudin-5 antibodies, mean pixel intensity was quantified using Fiji–ImageJ software (integrated intensity; threshold 80–180) and expressed as relative to cell numbers (DAPI staining). A minimum of five fields with ~30 cells/field from three separate differentiations were quantified and all experimental groups remained blinded until completion of the study. All images are representative images.

### Transwell co-culture

Twenty four- and 96-well Transwells^®^ (Corning^®^ 0.4 μm pore; Sigma–Aldrich) were coated with collagen IV/fibronectin on the apical side and 0.1% gelatin on the bottom well. hiPSC–BEC were dissociated and the seeded onto the apical side (3 × 10^5^/well); hiPSC–mural cells (MC) were dissociated and seeded onto the bottom well (25 × 10^4^/well). The next day, Transwells^®^ with BEC and MC were maintained without any further medium changes for 4 days before analyses.

### BEC functional assays

*TEER.* Transendothelial electrical resistance (TEER) measurements were taken every 24 h at day 6 of subculture of BEC onto Transwells^®^ using an EVOM2 Voltohmmeter/STX2 electrodes (World Precision Instruments). TEER was expressed as max mean.

*NaFl.* At 2 days post-subculture of BEC onto 24- and 96-well Transwells^®^, spent media was removed from the upper chamber of the Transwell^®^ and replaced with 600 µl of Sodium Fluorescein (NaFl, 1 mg/ml; Sigma–Aldrich) diluted 1:100 in endothelial serum-free media with B27. Samples of 100 µl were taken from the basolateral side every 2 h for a 6 h time course for 24-well Transwells^®^ and after 6 h for 96-well Transwells^®^. Raw fluorescence was measured with a FLUOstar Omega plate reader (excitation wavelength of 460 nm and emission 515 nm). Quantification was represented as percentage of total fluorescence at 6 h relative to empty coated Transwell^®^ well (blank) as previously described.

### MMPi treatments

hiPSC-derived BEC and mural cells were treated with DMSO (control), 13 MMP inhibitors (MMPi; 1 µM; Table 2); and doxycycline (5 µM; Sigma–Aldrich), with media change every other day for 2 days, and then harvested for analyses. cSVD iPSC–BEC lines were plated in triplicate in 96-well transwell plates on day 7 of differentiation. The following day, the media was replaced with DMSO-containing media for untreated samples (DMSO), MMP inhibitor-treated media or doxycycline (DOXY). The transwells were treated for 4 days, with media changes every 2 days. After 4 days, the media was replaced with NaFl-containing media, and the samples were incubated for 6 h. Signal-to-background ratio (S/B) is the ratio of the mean signal level to the mean background level. The *Z*′-factor is calculated as follows:

$$Z\prime \;=1-(3\sigma_{C+}+3\sigma_{C-})/|\mu_{C+}-\mu_{C-}|$$

**Table 2.** MMPi library.

Compound ID	Synonym	Target
A1	Ilomastat	MMP1, MMP2, MMP3, MMP9
B1	Marimastat	MMP1, MMP2, MMP3, MMP7, MMP14
C1	UK 356618	MMP1, MMP2, MMP3, MMP9, MMP13, MMP14
D1	S 3304	MMP9
E1	UK-370106	MMP2, MMP3, MMP7, MMP8, MMP9, MMP12, MMP13, MMP14
G1	ARP-100	MMP1, MMP2, MMP3, MMP7, MMP9
H1	PF-00356231 hydrochloride	MMP3, MMP8, MMP9, MMP12, MMP13
A2	PD-166793	MMP1, MMP2, MMP3, MMP9, MMP12, MMP13
B2	Tanomastat	MMP2, MMP3, MMP9, MMP13
C2	CP-471474	MMP1, MMP2, MMP3, MMP9, MMP13
D2	CP-544439	MMP1, MMP2, MMP3, MMP8, MMP9, MMP12, MMP13, MMP14
E2	Aderbasib	ADAM10, ADAM17
F2	Cipemastat	MMP1, MMP2, MMP3, MMP8, MMP9, MMP13

where σ_C+_ and σ_C−_ are the standard deviations of the positive and negative control groups, respectively, and μ_C+_ and μ_C−_ are the means of the positive and negative control groups.

Based on data from our preliminary studies, we calculated a mean difference of 17 and a standard deviation of 12 for our in vitro BBB permeability assay. Using these values, we determined that a sample size of *n* = 4/group (with biological triplicates) would be required to achieve 80% power at a significance level of 0.05. Thus, we used two disease lines for each of the SVD mutations each performed in triplicate.

### Annexin V apoptosis assay

Cells/ml 1 × 10^6^ were harvested and resuspended in 1× annexin-binding buffer and incubated with 5 μl of Annexin V–488 (Alexa Fluor 488 Annexin V/Dead Cell Apoptosis Kit; Life Technologies) for 15 min at room temperature. Cells were then resuspended in PBS with the addition of propidium iodide (PI, 1:300) and measured with a BD LRSFortessa Flow cytometer. Flow cytometric data were analysed with FCSalyzer 0.9.15-alpha software.

For 96-wells assay, the RealTime-Glo™ Annexin V Apoptosis Assay (Promega) was performed according to the manufacturer’s protocol. In brief, the cells (20,000 cells/well) were cultured in solid white bottom 96-well plates (Costar^®^ 3917) and treated with DMSO or MMP inhibitors. The prepared 2× Detection Reagent that contained Annexin V NanoBiT™ Substrate, Annexin V-SmBiT, Annexin V-LgBiT and CaCl_2_ was added in each well and the plates were shaken for 30 s at 500 rpm. The luminescence (RLU) readings were recorded at different time points (0, 2, 6, 12, 24 and 48 h post-treatment) using FLUOstar Omega plate reader. Background signals (from no-cell wells) that were subtracted from the wells that contained cells. Values are representative of 6-h measurements, which showed the most pronounced differences between disease and control cell lines (Figure S6).

### Statistical analysis

Data, expressed as means ± SD, were analysed statistically using SPSS 22.0 software. One-way or two-way ANOVA followed by LSD multiple comparisons was performed using GraphPad Prism 9.00 (GraphPad Software, Inc.) to analyse the significant difference, which was indicated as ns (not significant), *p* > 0.05; *p < 0.05; **p < 0.01; ***p < 0.001; ****p < 0.0001. The ‘*n*’, noted in the figure legends, represents the replicated number of biological experiments (independent differentiations). All data are representative of at least three independent experiments.

## Results

The following iPSC lines were successfully created: two cSVD lines with *HTRA1* mutations (R166H and P331L) and two with *COL4A1* (G755R) and *COL4A2* (G702D) mutations. In addition, we created isogenic gene edited iPSC clones for the *COL4A1/A2* and *HTRA1* mutations, reverting the mutation to the normal ‘unaffected’ variant. We also used four commercially available healthy (WT) iPSC lines.

### Creation of the iPSC lines and transwell models

All lines (Tables 1, S1 and S2)^10^ were characterised for pluripotency markers expression (Figure S1(a)–(c)).^10^ The lines were successfully differentiated into brain-endothelial like cells (BEC) and mural cells (MC). iPSC-derived BEC and MC were characterised for endothelial and MC markers expression, respectively, by RT-qPCR (Figure S2(a) and (b)). The cells were co-cultured in a transwell setting for phenotypic and functional analyses (Figure 1(a)).

**Figure 1.**
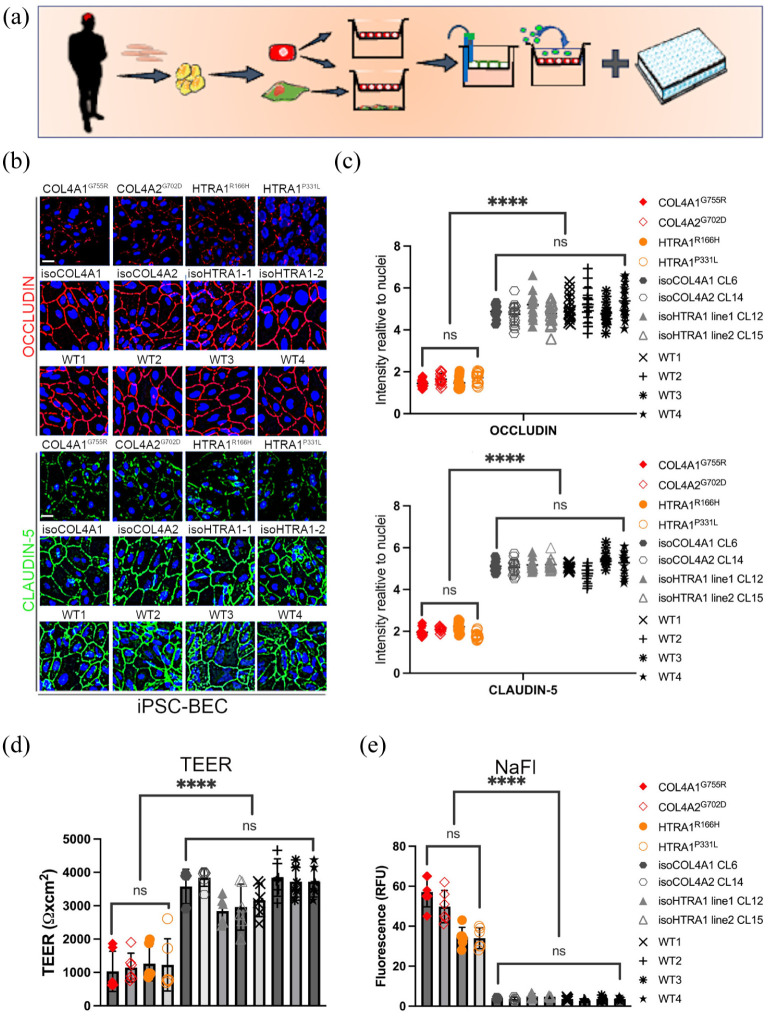
(a) Schematic illustrating the development of a transwell model of the BBB derived from cSVD patients, showcasing its applications in functional permeability studies and screening assays. (b) Junctional staining for occludin (red) and claudin-5 (green) in iPSC-derived BEC disease lines (COL4A1G755R, COL4A2G702D, HTRA1R166H, HTRA1P331L), isogenic lines (isoCOL4A1, isoCOL4A2, isoHTRA1-1, isoHTRA1-2) and healthy lines (WT1, WT2, WT3, WT4) cultured as monolayer. Nuclei were stained with DAPI; scale bar = 20 μm. (c) Quantification of total fluorescence intensity levels relative to cell numbers (nuclei) show significantly lower levels in disease lines compared to controls (biological replicates *n* = 3; five fields for each replicate for a total of 15 images/sample). (d) TEER peak values expressed as resistance (Ω) × cm^2^ for iPSC-derived BEC culture alone on top transwell show lower values in disease lines compared to controls (biological replicates *n* = 6). E) Size-selective NaFl permeability assay in transwell setting show higher permeability in disease lines measured after 6 h (biological replicates *n* = 6; Figure S3(b)). NaFl: sodium fluorescein; ns: not significant; RFU: raw fluorescence unit; TEER: transendothelial electrical resistance. The results are presented as means ± SD of *n* independent experiments. Statistical analysis was performed by two-way ANOVA with Tukey’s multiple comparison test. ****p < 0.0001.

### Identification of a common cSVD phenotype with increased permeability

To characterise the cellular phenotype of cSVD and identify common features, iPSC-derived brain endothelial cells (iPSC-BEC) were stained for occludin and claudin-5 (Figure 1(b)). Results showed a significant decrease in total fluorescence levels of both occludin and claudin-5 in cSVD iPSC-BECs compared to controls (Figure 1(c)).

To assess the functional impact of mutations on the blood–brain barrier (BBB), transendothelial resistance (TEER) measurements were performed using transwell systems, where iPSC–BECs were plated in the upper chamber. A common phenotype of increased permeability was seen across all lines derived from patients with *HTRA1* and *COL4A1/2* mutations with TEER values being consistently lower compared to the isogenic and wild type controls (Figures 1(d) and S3(a)). This increase in permeability was further validated by a size-selective permeability assay using sodium fluorescein (NaFl), which demonstrated increased permeability in all the disease lines compared to controls (Figures 1(e) and S3(b)).

In addition to the endothelial cell defects, across all disease lines we found abnormalities in the mural cells. Compared to controls, mutant cells showed an increase in actin stress fibers (Figure 2(a) and (b)) and increased apoptosis (Figure 2(c) and (d)). When disease iPSC–MC, but not control mural cells, were added to the basal side of the transwell, BBB permeability increased further (Figure 2(e)),^10^ demonstrating that across all disease lines mural cells play a role in mediating the increased BBB permeability. This effect is likely mediated by dysregulated matrix structure in *COL4A1/2* mutations and/or impaired ECM signalling pathways, such as TGF-β, in *HTRA1* mutations.

**Figure 2.**
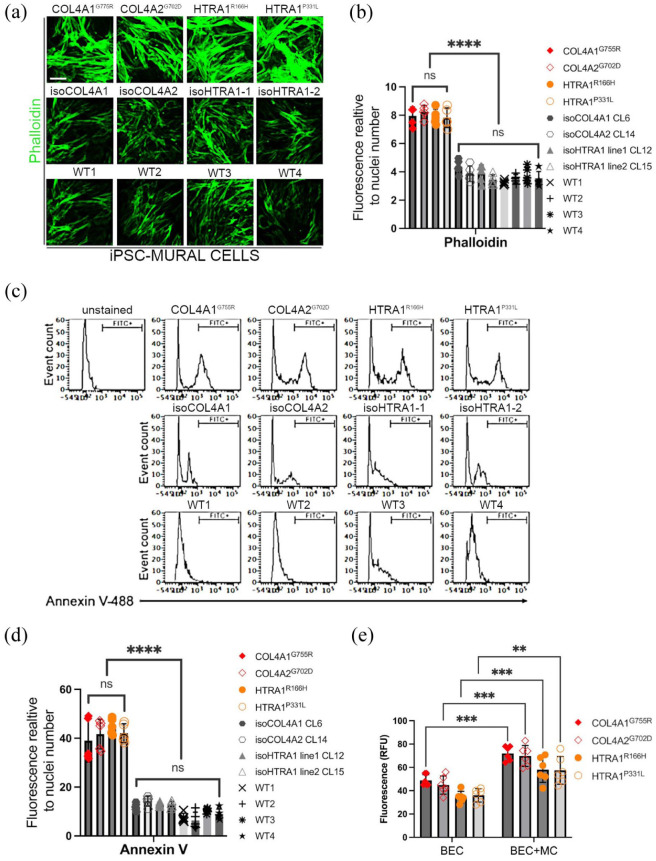
(a) Phalloidin staining for iPSC-derived MC and (b) quantification of total fluorescence relative to cell numbers (nuclei) shows increased levels in disease lines (COL4A1G755R, COL4A2G702D, HTRA1R166H, HTRA1P331L) compared to isogenic lines (isoCOL4A1, isoCOL4A2, isoHTRA1-1, isoHTRA1-2) and WT lines (WT1, WT2, WT3, WT4). Nuclei were stained with DAPI; scale bar = 50 μm. (c) Representative chart of flow cytometric analysis of annexin V-488 in iPSC–MC. (d) Quantification of the annexin V positive cells shows higher apoptotic rate in mutant lines compared to control MC lines (biological replicates, *n* = 6). (e) iPSC-derived disease BEC co-culture with iPSC-derived disease MC plated on the bottom of the transwell, shows increased BBB permeability compared to BEC alone transwell setting (biological replicates, *n* = 6). MC: mural cells; NaFl: sodium fluorescein; ns: not significant; RFU: raw fluorescence unit. The results are presented as means ± SD of *n* independent experiments. Statistical analysis was performed by two-way ANOVA with Tukey’s multiple comparison test. **p < 0.01. ***p < 0.001. ****p < 0.0001.

Our previous work suggested that mural cells may contribute to BBB defects through elevated expression of MMPs in *COL4A1/2* models^10^ (Figure 3(a)). We replicated this finding in the HTRA1 cSVD models (Figure 3(b)). We identified a subset of MMPs upregulated in our models, including collagenases (MMP1, MMP13), gelatinases (MMP2, MMP9), stromelysins (MMP3, MMP7) and others such as MMP12 and MMP14. Some of these MMPs were also upregulated in cSVD iPSC–BECs (Figure S4). Treatment with doxycycline (DOXY), a pan-MMP inhibitor, improved BBB properties in all disease lines, as evidenced by increased TEER and reduced NaFl permeability across all cSVD lines compared to untreated control (CTL; Figure 3(c) and (d)).

**Figure 3.**
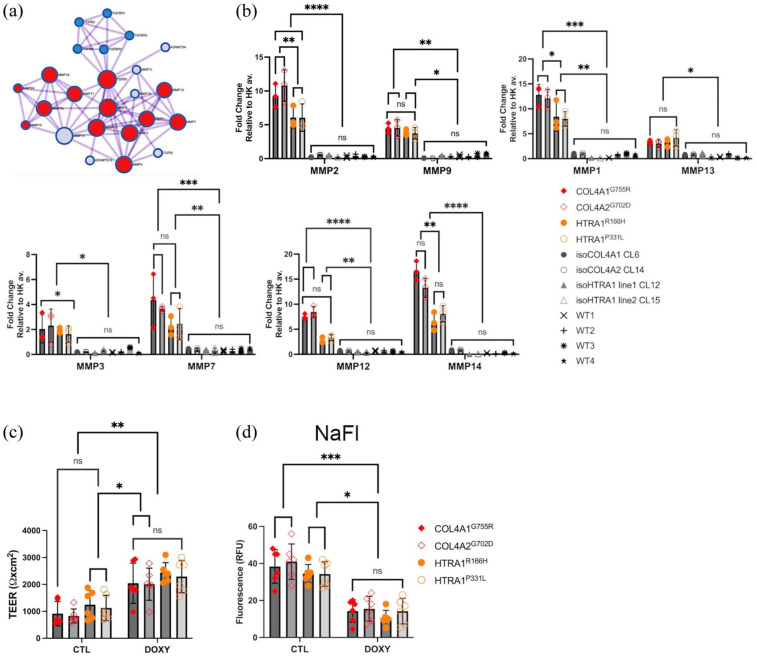
(a) Protein–protein interaction analysis by metascape shows enrichment for MMPs in disease COL4A1/2 iPSC-derived MC compared to isogenic lines by transcriptome (FDR of 5%).^10^ (b) RT–qPCR analysis performed at iPSC-derived MC shows significant increased levels for several MMPs, including MMP1, MMP2, MMP3, MMP7, MMP9, MMP12, MMP13 and MMP14 (biological replicates, *n* = 6). Disease iPSC–BBB models (COL4A1G755R, COL4A2G702D, HTRA1R166H, HTRA1P331L) upon treatment with DMSO (CTL) or doxycycline (5 µM; DOXY) for 4 days, were analysed for (c) TEER and (d) size-selective permeability NaFl assay, show increased TEER values and reduced permeability, respectively, upon DOXY treatment (biological replicates, *n* = 6). CTL: control; MMPs: matrix metalloproteinases; NaFl: sodium fluorescein; ns: not significant; TEER: transendothelial electrical resistance. The results are presented as means ± SD of *n* independent experiments. Statistical analysis was performed by two-way ANOVA with Tukey’s multiple comparison test. *p < 0.05. **p < 0.01. ***p < 0.001. ****p < 0.0001.

Taken together these data established a common shared molecular phenotype including tight junction defects with increased BBB permeability and elevated MMP levels that contribute to this BBB permeability.

### Creation and validation of a drug screening system

Given the shared phenotypes across cSVD models, and the ability to rescue these phenotypes with doxycycline treatment, we set out to determine if we can scale up our model for drug screening using BBB permeability as a readout. As proof of concept, we tested a small library of MMP inhibitors (MMPi) consisting of 13 small molecule inhibitors (Table 2).

The redout was taken after 6-h incubation period based on prior data showing significant differences in permeability between disease and control iPSC-BBB models at this time (Figure S3(b)). Controls (CTL) included both the four wild type lines and four isogenic lines. The media from the lower chamber was transferred to black 96-well plates, and fluorescence readings were taken using a plate reader (Figure 4(a)). The results were displayed as raw fluorescence units (RFU) relative to blank controls, presented as box plots (min to max; Figure 4(b)) and a heatmap by gene (Figure S5). The calculated *Z*′ score was slightly above 0.5, indicating that the assay is reliable.

**Figure 4.**
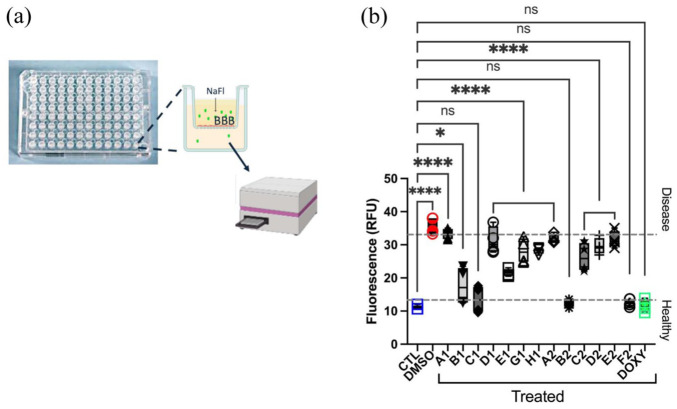
(a) Schematic of a 96-well transwell plate platform for fluorescence-based permeability assays. (b) Total fluorescence values relative to blank (well without cells) were plotted for control (blue; CTL; isoCOL4A1, isoCOL4A2, isoHTRA1-1, isoHTRA1-2, WT1, WT2, WT3, WT4), DMSO treated disease models (red), doxycycline treated disease models (green; DOXY) and MMP inhibitors (MMPi; A1, B1, C1, D1, E1, G1, H1, A2, B2, C2, D2, E2, F2; technical replicates, *n* = 3; biological replicates, *n* = 6). *Z*-score (*Z*′) = 0.50360; S/B ratio = 3.028136. Results show decrease levels of permeability of the disease lines to levels comparable to CTL and DOXY treated for C1, B2 and F2. ns: not significant; S/B: signal-to-background. The results are presented as means ± SD of *n* independent experiments. Statistical analysis was performed by two-way ANOVA with Tukey’s multiple comparison test. *p < 0.05. ****p < 0.0001.

We divided the range into healthy permeability (comparable to control WT and isogenic lines) and disease permeability (as seen in untreated disease iPSC–BEC, DMSO-treated). Most MMPi-treated samples showed permeability between the healthy and disease ranges, with three inhibitors (C1 = UK356618; B2 = Tanomastat; and F2 = Cipemastat) successfully reversing permeability to levels similar to controls and doxycycline-treated samples (Figure 4(b)). Similar responses to MMPi were seen across cell lines containing mutations in *HTRA1* and *COL4A1/2* genes.

We also determined if the MMP inhibitors could rescue other aspects of the cSVD model phenotype, including tight junction abnormalities and the increased cell death rates observed in MCs. The results showed that the three MMP inhibitors (UK 356618, Tanomastat and Cipemastat) effectively increased occludin and claudin-5 levels to values comparable to those observed in doxycycline-treated samples and controls (Figure 5(a) and (b)). Additionally, MMPi treatment significantly reduced the cell death rate in iPSC-derived cSVD MCs (Figures 5(c) and S6).

**Figure 5.**
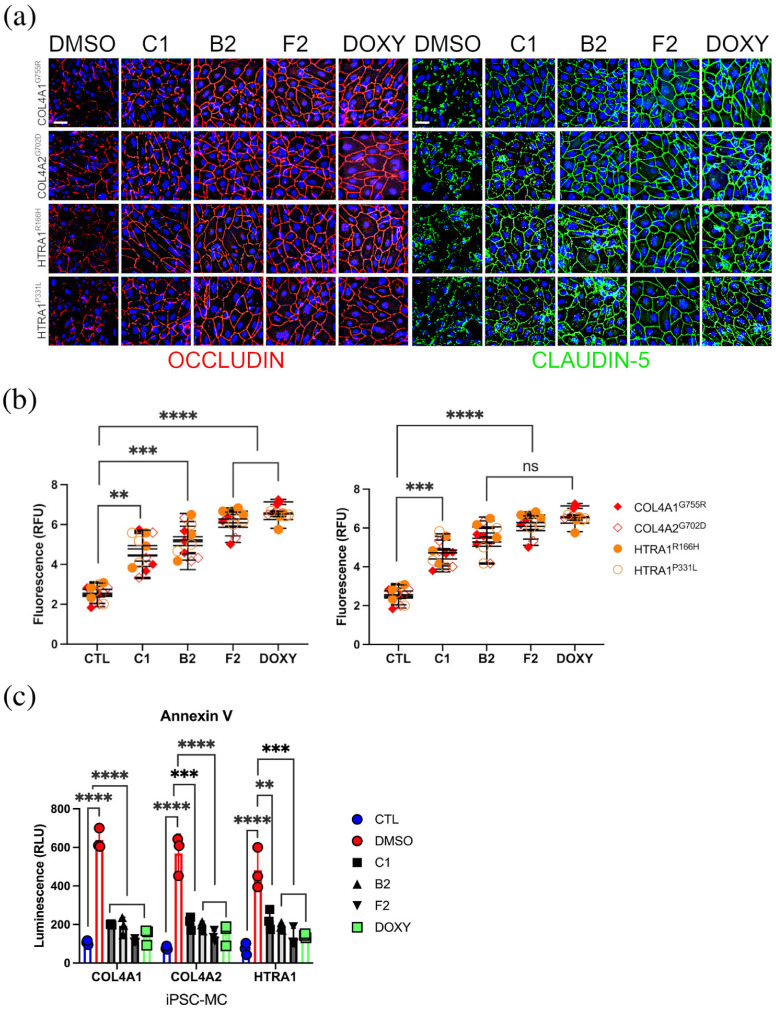
(a) Junctional staining for occludin (red) and claudin-5 (green) in iPSC-derived BEC disease lines (COL4A1G755R, COL4A2G702D, HTRA1R166H, HTRA1P331L) treated with DMSO or MMP inhibitors (C1, B2, F2) or with DOXY. Nuclei were stained with DAPI; scale bar = 20 μm. (b) Quantification of total fluorescence intensity levels relative to cell numbers (nuclei) upon treatment show significantly higher levels in disease lines treated with MMPi and DOXY compared to untreated lines (DMSO; biological replicates, *n* = 3; five fields for each replicate for a total of 15 images/sample). (c) RealTime-Glo™ Annexin V Apoptosis Assay was performed on iPSC-derived disease MC lines and the luminescence (RLU) readings were recorded at different time points (0, 6, 12, 24 and 48 h post-treatment; Figure S6; biological replicates, *n* = 3). RLU expressed as peak values show increased values for untreated disease lines (red; DMSO) and significantly reduced values for treated MC for MMPi (C1, B2 and F2) and DOXY to levels comparable to controls (blue; CTL; isogenics and WTs). *Z*-score (*Z*′) = 0.50579; S/B ratio = 6.185714, C1 = UK356618, B2 = Tanomastat, F2 = Cipemastat. DOXY: doxycycline; ns: not significant; S/B: signal-to-background. The results are presented as means ± SD of *n* independent experiments. Statistical analysis was performed by two-way ANOVA with Tukey’s multiple comparison test. **p < 0.01. ***p < 0.001. ****p < 0.0001.

These data establish that these cell models can be used in drug screening projects and MMPs hold potential as potential treatment targets across two forms of genetic cSVD.

## Discussion

In this study we demonstrated, using in vitro models derived from patient-derived iPSC, that monogenic cSVD caused by mutations in different genes (*COL4A1, COL4A2* and *HTRA1)* exhibit a common phenotype characterised by increased BBB permeability, disrupted tight junction integrity and degeneration of mural cells. We further demonstrate that such an in vitro model can be used to screen for drug therapies for cSVD and demonstrate as an exemplar the effectiveness of MMP inhibitors in reversing the disease phenotype across cells lines derived from patients with mutations in *COL4A1/A2* and *HTRA1* genes.

Our findings are consistent with monogenic forms of cSVD sharing convergent pathological mechanisms affecting the neurovascular unit (NVU). Despite being caused by mutations in different genes and the existence of distinct molecular mechanisms, a key shared pathophysiological mechanism appears to be ECM dysregulation. This disruption appears to be central to the breakdown of NVU function, particularly in the BBB, which is crucial for maintaining brain homeostasis. The results emerging from our in vitro models reinforce the view that disturbances in ECM homeostasis are a critical factor in the pathogenesis of cSVD, irrespective of the specific gene mutation involved.^7^

Specifically regarding *HTRA1*, mutations typically result in loss of serine protease function, impairing ECM turnover and leading to matrix accumulation.^12^ This defective ECM degradation may disrupt mural cell-ECM interactions, promoting mural cell dysfunction and apoptosis. Additionally, *HTRA1* acts as a negative regulator of TGF-β signalling; its loss may therefore lead to sustained TGF-β activation, which is known to induce MMP expression.^11,22,23^ The increased MMP production observed in HTRA1-mutant mural cells could thus represent a compensatory, but ultimately dysfunctional, response to ECM accumulation and excessive TGF-β activity. Together, these mechanisms could explain how *HTRA1* mutations contribute to mural cell pathology, elevated MMP levels and subsequent BBB compromise in our model.

A major limitation in drug discovery in cSVD has been a lack of clinically relevant in vitro models allowing drug screening. We demonstrated that our model system could be adapted to 96-well plates, and further development could allow even more compounds to be evaluated concurrently in a mid- or high throughput set up. By utilising these models, we were able to assess the impact of multiple inhibitors across various cSVD mutations using different functional readouts, including BBB permeability and mural cell apoptosis. As an example, we evaluated the role of MMP inhibition across all two disease models. MMPs have a well-established role in ECM remodelling and have been previously implicated in the pathophysiology of cSVD and stroke.^24^ In this study, we demonstrated that MMPs are consistently upregulated across different cSVD models, providing further evidence of their central role in BBB breakdown in cSVD. By utilising a small library of 13 MMP inhibitors, we successfully identified three inhibitors – UK356618, Tanomastat and Cipemastat – that effectively restored BBB integrity. These inhibitors demonstrated comparable efficacy to doxycycline, a pan-MMP inhibitor that we previously validated in our system.^10^ Interestingly, the effectiveness of specific MMP inhibitors varied depending on the mutation. For instance, Marimastat (B1) exhibited pronounced efficacy in COL4A1/2 mutant lines, which possibly could be attributed to the higher expression levels of MMP14 and MMP7 seen in cell lines containing these mutations.

Our results demonstrate shared disease mechanisms across different monogenic forms of cSVD. A key next step is to determine whether similar phenotypes can be observed in iPSCs from patients with sporadic cSVD. Increasing evidence suggests that NVU and ECM dysfunction also plays a key role in sporadic cSVD.^25^ Common genetic variants in *COL4A2* and *HTRA1*, genes causing monogenic cSVD, are associated with an increased risk of sporadic cSVD.^13,14,26^ If this could be demonstrated, it would make the results obtained from this model also applicable to the much larger burden of common sporadic cSVD. Future studies can build on this work by investigating shared mechanisms between monogenic and sporadic cSVD, potentially identifying common therapeutic targets.

Our 96-well format screening platform does not qualify as high-throughput screening (HTS) by conventional standards. However, the results, including a *Z*′ score above 0.5, suggest that this platform could be optimised and scaled for HTS applications as previously shown.^27^ With further refinement, this system holds potential for rapid and cost-effective testing of therapeutic candidates, making it a valuable tool in drug discovery. Additionally, the ability to model multiple cSVD mutations simultaneously highlights the versatility of this approach for screening both small-molecule inhibitors and novel therapies. While this model offers a method to assess the efficacy of potential treatments, it should not be used in isolation. It is best integrated as one component of the broader drug development pipeline. Further validation in animal models is essential to confirm efficacy, assess toxicity and pave the way for eventual evaluation in humans.

Although we demonstrated efficacy of MMP inhibitors in restoring BBB integrity in vitro, there are potential limitations to the application of MMP inhibitors in humans. Many MMP-targeting drugs currently available exhibit significant toxicity, limiting their clinical utility.^28^ Although doxycycline, an antibiotic medication used for many years in humans, show efficacy in rescuing the BBB phenotype in our models, and has been shown to protect against brain injury in a rodent model of cSVD,^29^ it had no effect on reducing MRI measured BBB permeability in a phase 2 trial in humans.^30^ One possible factor in this species difference may be the much higher dose given in animal models. Moreover, while our model has demonstrated the efficacy of MMP inhibitors in restoring BBB integrity, the translation of these findings to human disease remains uncertain, given the challenges with toxicity and efficacy seen in clinical trials of MMP inhibitors. Further in vivo studies and clinical validation will be crucial for confirming the therapeutic relevance of our findings.

While our iPSC-based model provides valuable insights into the pathophysiology of monogenic cSVD and offers a powerful platform for drug screening, there are inherent limitations to its use. One primary limitation is that this model represents an in vitro system, which may not fully capture the complexity of the human brain’s in vivo environment, including the interactions between systemic factors, immune responses and the broader vascular network. Another challenge is the variability in differentiation potential of iPSCs, which can introduce batch effects and differences between cell lines. This variability necessitates careful experimental controls and validation in future studies.

In conclusion, our study demonstrates that iPSC models for two monogenic forms of cSVD, Gould Syndrome and CADASIL2, share common phenotypic features and highlights key involvement of BBB permeability and ECM disruption. Our iPSC model offers a promising platform for the screening of therapeutic candidates.

## Supplemental Material

sj-docx-1-jcb-10.1177_0271678X251389379 – Supplemental material for An iPSC-derived model for drug screening in cerebral small vessel disease Supplemental material, sj-docx-1-jcb-10.1177_0271678X251389379 for An iPSC-derived model for drug screening in cerebral small vessel disease by Alessandra Granata, Maha Al-Thani, Mary Goodwin-Trotman, David M Smith, Tom Van Agtmael and Hugh S Markus in Journal of Cerebral Blood Flow & Metabolism
