# Supplementary material for: An iPSC-derived model for drug screening in cerebral small vessel disease
Source: J Cereb Blood Flow Metab. 2025 Nov 7;46(6):1480–92. doi: 10.1177/0271678X251389379 (PMC12597805; doi:10.1177/0271678X251389379)
Supplement: Supplementary file 1 [file sj-docx-1-jcb-10.1177_0271678X251389379.docx]

**Table S1. *HTRA1* line 1 (R166H) and line 2 (P331L) hiPSC characterisation and validation.**

| **Classification** | **Test** | **Result** | **Data** |
| --- | --- | --- | --- |
| Morphology Phenotype | Photography Qualitative analysis: Immunocytochemistry performed by the Cambridge Biomedical Research Centre iPSC core | Positive for OCT4, NANOG, SOX2, TRA-1 | **Figure S1A** |
|  | Quantitative analysis: qPCR performed by the Cambridge Biomedical Research Centre iPSC core | Expression of *OCT4, NANOG, c-Myc* | **Figure S1B** |
| Differentiation potential | Trilineage potential performed by the Cambridge Biomedical Research Centre iPSC core | Positive for three germ layer markers: endoderm marker (GATA4, SOX17); mesoderm marker (EOMES, BRACHYURY) and ectoderm marker (SOX1, NESTIN) | **Figure S1C** |
| Genotype | Karyotype performed by the Medical Genetics Service Cytogenetics Laboratory <https://www.cuh.nhs.uk/our-services/genomic-laboratory/> | XX, 46  Normal at passage 22 | **Figure S1D** |
| Mutation analysis | Sanger Sequence | c.947 G>A p.R166H;  c.992 C>T p.P331L | **Figure S1E** |
| HepB/ HepC/HIV/ Mycoplasma Test | h-IMPACT Profile III (1-2) performed by IDEXX BioResearch  [www.idexxbioresearch.com/europe](http://www.idexxbioresearch.com/europe) | All test results were negative |  |

**Table S2. CRISPR sgRNA guide and ssODN** **related to Figure S1.**

| Gene/Mutation | Sequencing primers sequence 5’-3’ | gRNA | Donor sequence 5’-3’ (ssODN)  Corrected base  Mutated PAM |
| --- | --- | --- | --- |
| *HTRA1^R166H^* | ACTCCGCCTTCAGAATTCCA | ATTTATATTGAAATAGCGCC | TCTTTGCTTTTGTTCTCAGGGCAGGAAGATCCCAACAGTTTGCGTCATAAATATAACTTTATCGCGGACGTGGTGGAGAAGATCGCCCCT |
| *HTRA1^P331L^* | CCCTGTTTACGGCACCTCTA | TTAATTGCAGTATGGAAACT | CTTTTCATTTCTGTTTAATTGCAGTATGGAAACTCAGGAGGCCCGTTAGTAAACCTGGTAAGGTCTTTTAAACCTATGTTAGGTCATTT |

**Table S3. Quantitative real-time PCR Primers** **set used in this study, related to Figure 3, S1, S2, S4).**

| Gene Target | Forward Sequence 5’-3’ | Reverse Sequence 5’-3’ |
| --- | --- | --- |
| *GAPDH* | AACAGCCTCAAGATCATCAGC | GGATGATGTTCTGGAGAGCC |
| *HMBS* (PBGD) | GGAGCCATGTCTGGTAACGG | CCACGCGAATCACTCTCATCT |
| *POU5F1* (OCT4) | AGGGCAAGCGATCAAGCA | GGAAAGGGACCGAGGAGTA |
| *SOX2* | ATGCACCGCTACGACGTGA | CTTTTGCACCCCTCCCATTT |
| *c-MYC* | AGGCGAACACACAACGTCTT | TTGGACGGACAGGATGTATGC |
| *NANOG* | ACTAACATGAGTGTGGATCC | TCATCTTCACACGTCTTTCAG |
| *PECAM1* | CAGGCGCCGGGAGAAGTGAC | CGTCCAGTCCGGCAGGCTCT |
| *CD34* | CACAGGAGAAAGGCTGGGCGA | TGGCCGTTTCTGGAGGTGGC |
| *OCLN* | GGAGTGAACCCAACTGCTCA | CTCCTGGGGATCCACAACAC |
| *CDH5* | GGTCAAACTGCCCATACTTG | CGCAATAGACAAGGACATAACAC |
| *CLDN5* | CAGTACCGCAGGAAGAGGAG | ATCCCATGGCAAACAGAGAG |
| *OCLN* | GCTTGCAAACTGCTTCAGCT | AGAAAGGCAGAATCCCAGGC |
| *MMP1* | CTGGCCACAACTGCCAAATG | CTGTCCCTGAACAGCCCAGTACTTA |
| *MMP2* | TCTCCTGACATTGACCTTGGC | CAAGGTGCTGGCTGAGTAGATC |
| *MMP3* | CTCACAGACCTGACTCGGTT | AAAGCAGGATCACAGTTGGC |
| *MMP7* | CATGAGTGAGCTACAGTGGGA | CTATGACGCGGGAGTTTAACAT |
| *MMP9* | TTGACAGCGACAAGAAGTGG | GCCATTCACGTCGTCCTTAT |
| *MMP12* | GTTCCTCACTGCTGTTCACG | GCGTTGGTTCTCTTTTGGGT |
| *MMP13* | TTTCAACGGACCCATACAGTTTG | CATGACGCGAACAATACGGTTA |
| *MMP14* | CAGAGAAGGCACACAAACGA | CACTGGTGAGACAGGCTTGA |
| *CNN1* | GTCCACCCTCCTGGCTTT | AAACTTGTTGGTGCCCATCT |
| *ACTA2* | ACAAGACCTCATAGCCAGCAC | CTGTTGGCTCCTTGCTTGTTC |
| *TAGLN* | TCTTTGAAGGCAAAGACATGG | TTATGCTCCTGCGCTTTCTT |
| *MCAM* | AAGACCAAGATCCACAGCGA | TGGTTGTGTTGGAGTCTGGT |
| *CSPG4* (NG2) | TTCCAGCTGAGCATGTCTGA | TCCTCCCGATCTGAAACCAC |
| *PDGFRB* | GCTTAAATCCACAGCCCGCA | AGGTAGTCCACCAGGTCTC |

**Table S4. Primary antibodies** **list used in this study, related to Figure 1, 2, 5 and S1.**

| Target antigen | Species | Supplier | Catalogue n. | Use |
| --- | --- | --- | --- | --- |
| OCT3/4 | Mouse | Santa Cruz | SC-5279 | Immunofluorescence |
| SOX2 | Mouse | Abcam | sc-21705 | Immunofluorescence |
| NANOG | Goat | R&D | AF1997 | Immunofluorescence |
| TRA-1-60 | Mouse | Santa Cruz | sc-21705 | Immunofluorescence |
| GATA4 | Rabbit | Abcam | ab61767 | Immunofluorescence |
| SOX17 | Goat | R&D | F1924 | Immunofluorescence |
| EOMES | Rabbit | Abcam | ab23345 | Immunofluorescence |
| BRACHYURY | Mouse | Santa Cruz | sc-166962 | Immunofluorescence |
| SOX1 | Goat | R&D | AF3369 | Immunofluorescence |
| NESTIN | Mouse | BD Bioscience | 611659 | Immunofluorescence |
| Occludin | Mouse | Thermo Fisher | 331500 | Immunofluorescence |
| claudin-5 | Rabbit | Abcam | ab15106 | Immunofluorescence |
| Smooth Muscle Actin | Mouse | Agilent | M085101-2 | Immunofluorescence |
| SM22 | Rabbit | Abcam | ab14106 | Immunofluorescence |
| Calponin | Mouse | Sigma-Aldrich | C-2687 | Immunofluorescence |
| Annexin V-488 |  | Life technologies | V13241 | Flow cytometry |
| CytoPainter Phalloidin- 488 |  | Abcam | ab176753 | Immunofluorescence |

**Figure S1.** *HTRA1R166H* and *HTRA1P331L* hiPSC lines were characterised for: **A)** Pluripotency by immunostaining for OCT4, NANOG, SOX2, TRA-1-60 and SSEA4. scale bar=40μm; **B)** qRT-PCR expression of *POU5F1* (OCT4), *NANOG* and c-myc. **C)** Immunostaining analysis for each of the three germ layers (GATA-4 and SOX-17, endoderm; BRACHYURY and EOMES, mesoderm; NESTIN and SOX1, ectoderm); nuclei were stained with DAPI; scale bar=40μm. **D)** Karyotype analysis with normal karyotype, with 46 chromosomes (46, XX) identified in both hiPSC lines. **E)** Sanger sequencing output for *HTRA1* heterozygous mutations (Arg166His and Pro331Leu) and CRISPR-corrected isogenics. **F)** Immunostaining analysis for pluripotency markers (OCT3/4 and SOX2); nuclei were stained with DAPI (insert); scale bar=50μm; and **G)** Quantitative real-time PCR analysis for pluripotency markers expression (*POU5F1*, *SOX2* and *NANOG*) for isogenic lines (isoHTRA1-1, isoHTRA1-2). The results are presented as means ± SD of 3 biological replicates; ns (not significant). Statistical analysis was performed by 2-way ANOVA with Tukey’s multiple comparison test.

**Figure S2. A)** mRNA profile of iPSC-derived (brain endothelial like cells; BEC) by quantitative real-time PCR for specific markers (*CD34, PECAM-1, OCLN, CDH5 and CLDN5*) (biological replicates n=6) and **B)** Quantitative real-time PCR for smooth muscle cells markers (*CNN1, ACTA2, TAGLN, NG2* and *MCAM)* for iPSC-derived mural cells (MC) (biological replicates n=3) for iPSC disease lines: COL4A1G755R, COL4A2G702D, HTRA1R166H, HTRA1P331L; isogenic lines: isoCOL4A1, isoCOL4A2, isoHTRA1-1, isoHTRA1-2) and WT lines: WT1, WT2, WT3, WT4. The results are presented as means ± SD of n independent experiments; *P<0.05; ns (not significant). Statistical analysis was performed by 2-way ANOVA with Tukey’s multiple comparison test.

**Figure S3. A)** Representative transendothelial electrical resistance (TEER) 6 days time-course expressed as resistance (Ω) x cm^2^ for iPSC-derived disease, isogenic and WTs BEC. **B)** Representative size-selective sodium fluorescein (NaFl) permeability assay 6 hours time course for iPSC-derived disease, isogenic and WTs BEC. The results are presented as means ± SD of n = 3 biological replicates *P<0.05; **P<0.01; ***P<0.001; ns (not significant). Statistical analysis was performed by 2-way ANOVA with Tukey’s multiple comparison test.

**Figure S4.** mRNA profile of iPSC-derived BEC by quantitative real-time PCR for MMPs: MMP1, MMP2, MMP3, MMP7, MMP9, MMP12, MMP13 and MMP14. The results are presented as means ± SD of n = 6 biological replicates *P<0.05; **P<0.01; ***P<0.001; ns (not significant). Statistical analysis was performed by 2-way ANOVA with Tukey’s multiple comparison test.

**Figure S5.** Heatmap by gene for COL4A1G755R, COL4A2G702D, HTRA1R166H, HTRA1P331L iPSC-BBB treated with DMSO (control) or with MMP inhibitors (MMPi; A1, B1, C1, D1, E1, G1, H1, A2, B2, C2, D2, E2, F2) or doxycycline (DOXY). Control (CTL) = isogenics (isoCOL4A1, isoCOL4A2, isoHTRA1-1, isoHTRA1-2) and healthy (WT1, WT2, WT3, WT4).

**Figure S6.** Sequentially repeated measures of luminescence (RLU) with RealTime-Glo Annexin V Apoptosis assay in disease (COL4A1G755R, COL4A2G702D, HTRA1R166H, HTRA1P331L), isogenics (isoCOL4A1, isoCOL4A2, isoHTRA1-1, isoHTRA1-2) and healthy (WT1, WT2, WT3, WT4) iPSC-derived MC in a 96-well plate format (20,000 cells/well). RFU= raw fluorescence unit. Data present the mean of 3 readings for each line (biological replicates n = 3) ± SD *P<0.05; ****P<0.001; ns (not significant). Statistical analysis was performed by 2-way ANOVA with Tukey’s multiple comparison test.
